# Supplementary material for: The Heteroepitaxy of Thick β-Ga2O3 Film on Sapphire Substrate with a β-(AlxGa1−x)2O3 Intermediate Buffer Layer
Source: Materials (Basel). 2023 Mar 30;16(7):2775. doi: 10.3390/ma16072775 (PMC10095721; doi:10.3390/ma16072775)
Supplement: Supplementary file 1 [file materials-16-02775-s001.zip › materials-2296442-supplementary.pdf]

Figure S1 shows the β-Ga2O3 film thickness measured by cross-section SEM. The growth rate of β-Ga2O3 film is roughly estimated around 5.25, 6 and 4 μm/h according to the film thickness of 10.5, 12 and 8 μm, respectively, for grown on sapphire without buffer, with buffer by carbothermal reduction and HVPE, as shown in Figure 1(a), (b) and (c).

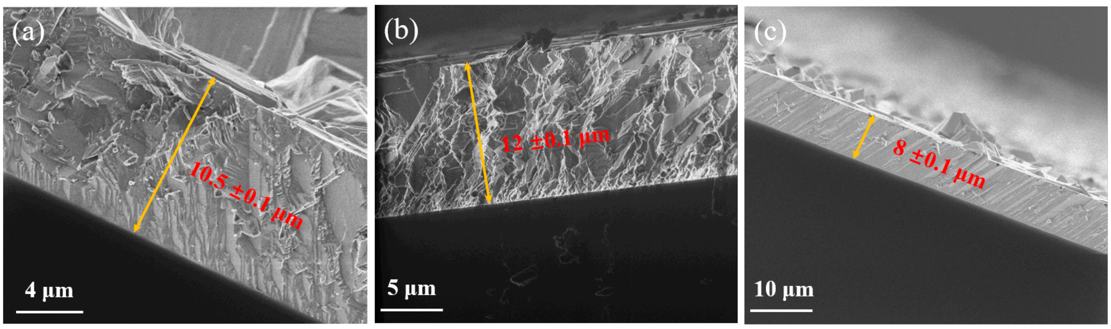

Figure S1. The cross-section SEM image of β-Ga_2O_3 thick film grown directly on sapphire substrate (a). The cross-section SEM image of β-Ga_2O_3 thick film grown on β-$(Al_{1-x}Ga_x)_2O_3$/sapphire substrate by carbothermal reduction (b) and HVPE (c) method.
